# Bath Workhouse Infirmary

**Published:** 1914-07-18

**Authors:** W. E. Winckworth

**Affiliations:** Clerk


### THE EDITOR'S LETTER-BOX.

### Bath Workhouse Infirmary.

*To the Editor of* The Hospital.

Sir,—I am directed by the Guardians of the Bath Union to forward you the following resolution which was unanimously adopted by them at their meeting on July 1, 1914.

Yours faithfully,
W. E. Winckworth, Clerk.

Resolution.

"That the attention of this board having been called to a statement in The Hospital to the effect: That the Infirmary at Frome Road House was in such bad repute amongst the sick poor that they preferred to die in their homes than enter it, strongly protests against that statement as being entirely untrue and grossly unjust to the staff at the infirmary, and instructs the Clerk to record this resolution upon the minutes and forward a copy of it to the Editor of the paper in question."

Bath Union, Union Offices, 3 North Parade, Bath,
July 10, 1914.

[We would point out to the Bath Guardians that the statement they attribute to us was made by Dr. Fuller at one of their own meetings. The protest should therefore have been addressed to Dr. Fuller. Dr. Fuller did not criticise the infirmary staff, but he did criticise very severely the structural arrangements at the infirmary and the Guardians for their neglect to remedy them. We might add that Mr. Court, the Local Government Board inspector, stated at the same meeting of the Guardians that the present wards were absolutely unfit in the winter for old people with bronchitis. The Guardians would be better occupied in remedying the admitted defects of the infirmary wards than in suggesting that their alleged bad repute amongst the Bath poor is a gross injustice to the infirmary staff.—Ed. The Hospital.]

